# Observations on the Nesting and Prey of the Solitary Wasp, *Tachysphex inconspicuus*, with a Review of Nesting Behavior in the *T. obscuripennis* species group

**DOI:** 10.1673/031.010.14143

**Published:** 2010-10-22

**Authors:** Frank E. Kurczewski, Rollin E. Coville, Coby Schal

**Affiliations:** ^1^ Post Office Box 15251, Syracuse, New York 13215, U.S.A; ^2^ 6201 Tehama Avenue, Richmond, California 94804, U.S.A; ^3^ Department of Entomology, Campus Box 7613, North Carolina State University, Raleigh, North Carolina 276957613, U.S.A

**Keywords:** Blattaria, canopy indicator, *Chorisoneura*, cockroach, *Riatia*, tropical wet forest

## Abstract

The nesting behaviors of 10 females of *Tachysphex inconspicuus* (Kirby) (Hymenoptera: Crabronidae) were studied on a sandy, mowed lawn at the La Selva Biological Station in northeastern Costa Rica on 27–29 April 1980. Twenty-four completed nests were observed, excavated, and measured. The nests had oblique, short burrows leading to one or two shallow cells. Prey cockroaches belonging to 11 species of *Chorisoneura* and *Riatia fulgida* (Saussure) (Blattaria: Blattellidae), all tropical wet forest canopy indicator species, were removed from the cells, weighed, and identified. The cockroaches consisted mainly of adult females, selectively preyed upon over adult males and nymphs due to their larger sizes. The aggregate prey mass in cells was separable into prospective larger (heavier) female and smaller (lighter) male cells. Wasps usually oviposited on the heaviest cockroach in a cell, in most cases an adult female. Atypical genus behavior included (1) prey being carried to one side of the wasp and perhaps grasped by a hindleg during removal of the temporary entrance closure and nest entry and (2) wasp's egg being laid affixed to a forecoxal corium and extending backward in a longitudinally posteriad position across the prey's ventral thorax. A comparison with the nesting behavior of other species in the *Tachysphex obscuripennis* species group is made.

## Introduction

*Tachysphex* is a very large, highly evolved and complex genus of small ground-nesting solitary wasps, with 445 species worldwide and 85 species in North America, Central America, and the Caribbean Region (Bohart and Menke 1976; Pulawski 1988, 2009). The species are mainly cursorial and fast moving (Williams 1914) as the genus name implies (tachys = swift, sphex = wasp). The North American, Central American, and Caribbean *Tachysphex* excavate mostly short burrows and shallow cells in sandy, gravelly, or (rarely) loamy soils. They stock their cells with grasshoppers (Acrididae), katydids (Tettigoniidae), crickets (Gryllidae), cockroaches (Blattaria), and mantids (Mantodea) (Krombein 1979; Kurczewski 1966, 2010; Pulawski 1974, 1988). There is even a single record of *T. apricus* Pulawski pinned with a nymphal walking stick (Phasmodea) more than four times its length (Elliott and Kurczewski 1985), although Pulawski (1988) questions its authenticity.

The *Tachysphex obscuripennis* species group is the only group known to prey on cockroaches (Evans et al. 1976; Krombein and Pulawski 1994; Kurczewski 2010; Pulawski 1971, 1974, 1988, 2007). This group has numerous species in the Afrotropical, Oriental, and Australasian Regions (Evans et al. 1976; Krombein and Pulawski 1994; Pulawski 1977, 2007). This group contains some of the more unusual species in the genus from the standpoint of morphology and behavior. The flattened scutum and scutellum of the female are apparently adaptations for hunting cockroaches, allowing the wasp to penetrate narrow crevices where the prey lives (Bohart and Menke 1976, Evans et al. 1976, Pulawski 2007). The stout, asymmetrical apical tarsal segments of the female with matlike vestiture on the ventral surface are probably adaptations for capturing and carrying the cockroach (Bohart and Menke 1976; Pulawski 2007). Some species in the *obscuripennis* group have fewer, shorter, wellspaced rake spines on the foretarsus than other *Tachysphex* (Bohart and Menke 1976; Pulawski 1988). Such a reduction in the foretarsal rake apparatus implies that these species may excavate their burrows in finergrained soils.

The *obscuripennis* group is poorly represented in the Nearctic, Caribbean, and Neotropical Regions with only three species: *Tachysphex inconspicuus* (Kirby) (Hymenoptera: Crabronidae), *T. iridipennis* (F. Smith 1873), and *T. alayoi* Pulawski 1974 (Bohart and Menke 1976; Pulawski 1988). *Tachysphex inconspicuus* and *T. iridipennis* are moderately common in tropical regions of Mexico, Central America, and South America (Pulawski 1974, 1988). *Tachysphex alayoi* is found mainly in the West Indies (Pulawski 1988). All species hunt and stock their cells with small adult and nymphal cockroaches belonging to the family Blattellidae (Pulawski 1974, 1988), although there is one record of a nymphal cricket as prey (Buys 2007).

Relatively little is known about the ecology, nesting behavior, nest structure, and prey of the American cockroach-hunting species in the *obscuripennis* group. Elliott et al. (1979) collected provisioning females of *T. alayoi* on inland soils in the Bahamas, the wasps having flown from higher vegetation to the ground with slightly larger cockroaches of the genus *Symploce.* Genaro (2004) reported *T. alayoi* females nesting in barren, coarse-grained beach sand in Cuba and transporting cockroaches directly into open burrows. One burrow was 11 cm long with a single cell, 5 cm below the surface. It held two lightly paralyzed prey: an adult female and immature *Cariblatta* sp., 6.7 and 5.5 mm long, respectively. One of the cockroaches had some of its antennal and tarsal segments cut off, possibly due to the interaction with the wasp during capture (Genaro 2004). The wasp's egg was affixed to the forecoxa of a prey and extended backward across its thoracic venter.

Rau (1933) noted a *T. iridipennis* or *T. inconspicuus* female flying with a partly paralyzed *Anaplecta asema* on Barrow Colorado Island in the Panama Canal Zone. Vesey-FitzGerald (1956) collected females of *T. iridipennis* in Trinidad, evidently nesting in beach sand and provisioning with adult *Cariblatta tobagensis.* Pulawski (1988) reported adults of *Euthlastoblatta abortiva* and *Ischnoptera rufa debilis* as prey of *T. iridipennis.*

Williams (1941) described *T. blatticidus*, having since been synonymized with *T. inconspicuus* (Pulawski 1974), so named for several cockroaches (Blattaria: Blattellidae) from Trinidad being pinned with the wasps. Callan (1942) reported the mutillid *Timulla eriphyla* cleptoparasitizing the cells of *T. blatticidus* (= *T. inconspicuus*) in Trinidad. Callan (1954, 1993) noted *T. inconspicuus* nesting in sandy soils of a sandpit in Trinidad and sand beach in Venezuela and provisioning with the blattellids *Chorisoneura fuscipennis, C. gemmicula*, and *Riatia orientis* (Trinidad), and *C.* sp. (Venezuela). Buys (2006) described the last instar larva of *T. inconspicuus*, reared from an adult *Chorisoneura excelsa* in southeast Brazil.

Buys (2007) provided the most complete observations to date on the nesting behavior of an American cockroach-hunting *Tachysphex.* He observed the nesting behavior of *T. inconspicuus* at two locations in southeast Brazil, a sand beach and a sand-patched dirt road. Buys (2007) reported that females leveled the sand removed from their excavations, temporarily closed their nest entrances with soil, and then made orientation flights above the nesting area. Lightly paralyzed cockroaches were consecutively brought to the nest predominantly in flight. They were grasped by their antennae with the wasp's mandibles and by their body with the wasp's legs. A female removed the sand closure from her entrance with the forelegs, retaining her grasp of the prey's body with a hindleg as she hurriedly entered the burrow.

Nests of *T. inconspicuus* excavated by Buys (2007) were single-celled. They had an oblique burrow that terminated in a cell 6–7 cm deep in the sand beach, but only 1–2.5 cm deep in the sand-patched dirt road. Two to four adult and nymphal *Chorisoneura*, nymphal *Riatia*, and a single nymphal gryllid were found in the completed cells or collected from provisioning females. The cockroaches were positioned in a cell ventral side upward. The wasp's egg was laid on the ventral thorax of one of the last prey taken into the nest (Buys 2007).

The contents of *T. inconspicuus* cells were cleptoparasitized at the sand beach location by a miltogrammine fly, *Amobia floridensis* (Townsend) (Sarcophagidae) (Buys 2007). The wasps dug 1–4 “accessory” holes around their entrances in the sand beach to dissuade the cleptoparasitic flies from attempting larviposition in the burrows.

Four females of *T. inconspicuus* (det. W. J. Pulawski) in the Smithsonian Institution are pinned atop their *Chorisoneura* prey (det. G. Beccaloni). They were hand-netted at Kartabo Point, Guyana from 22–24 December 1983 by Warren E. Steiner. The wasps are 6–7 mm and the cockroaches are 8–9 mm long. The wasps were nesting in bare, level “deep white sand” in “dry sunlit spots” surrounded by “secondary disturbed rainforest” (Steiner W. E. 2009 personal observation).

*Tachysphex inconspicuus*, the subject of this study, is the commonest and most widely distributed of the American species in the *obscuripennis* group (Pulawski 1974, 1988). Females are black and 6–8.5 mm long (Pulawski 1988). Females of *T. iridipennis* are also black, but larger in size (8–10.5 mm, Pulawski 1988). In addition to being smaller, females of *T. inconspicuus* can be readily separated from those of *T. iridipennis* by the much longer setae of the foretarsal digging rake (Pulawski 1988). Females of *T. alayoi* are highly variable in size (6–12 mm) and black with the last three abdominal segments reddish (Pulawski 1988).

This study on the nesting behavior of *T. inconspicuus* compliments rather than duplicates Buys' (2007) study. Whereas Buys' (2007) study concentrated on above ground activities, this study focused on the wasps' nests, prey species, prey size (weights), hunting locale, and characteristic egg placement. The first photographs of some aspects of the nesting behavior of *T. inconspicuus* are presented.

## Materials and Methods

Field observations on *T. inconspicuus* were made at the La Selva Biological Station in northeastern Costa Rica (10° 26′ N, 83° 59′ W). The aggregation nested in the soil beside the Old Station living quarters. Ten wasps and their nests were marked and observed 27–29 April 1980 in areas of exposed sandy soil in a level, mowed grassy lawn. Periodic rainfall interrupted some of the observations despite this period (February–April) being the driest of the year. The wasps were observed for approximately 12.5 h.

Additional collections of males and females of *T. inconspicuus* were made through 7 May 1980. Other visits to the station during March– June 1981, May 1986, September 1987, and December 1990 revealed no wasp activity at this site.

*T. inconspicuus* were timed during burrow excavation, temporary closure, orientation flight(s), hunting and prey transport, periodic returns to the nest with and without prey, and final closure. Certain individuals and their activities were photographed during aspects of the nesting sequence and contents of the cells they worked on were examined after the cell contents had been exhumed and sorted.

Twenty-four apparently completed nests were excavated during the three day study period. The dimensions of three nests and five cells were recorded and the nests sketched. In addition, prey taken from provisioning wasps and incomplete nests were collected. A total of 69 cockroaches were preserved at the station and later identified to species by Dr. Frank W. Fisk, the Ohio State University; Columbus, Ohio.

Burrow length, cell depth, and cell size (height, width, and length) were measured. The number of prey per fully provisioned cell, position of prey in the cell, and placement of the wasp's egg on the pedestal prey were recorded in the field. Prey were removed from the cells, placed in individual glassine envelopes according to nest and cell number, and weighed (wet). The aggregate prey weight of each fully provisioned cell was summed. Live eggs were measured under a microscope using an ocular micrometer.

Fresh aggregate and individual body mass (wet weight) of nymphs and adult males and females were subjected to analysis of variance (ANOVA, PROC GLM) in SAS 9.1 (SAS Institute 2004), followed by preplanned comparison of means using Fisher's least square difference (LSD, α = 0.05). Comparisons of the body mass of egg-bearing and non-egg-bearing prey within the same cell were done with the paired Student's *t*-test. Standard error of the mean is given with all means.

## Results

### Burrow excavation

Females began excavating their burrows with the mandibles. Once the mandibles loosened the surface soil, the forelegs were used to rake it backward beneath the synchronously lifting abdomen. The foretarsi bear a series of long, lateral spines that assist in moving the loosened sand grains. Females backed from their excavations at intervals to remove the loose sand that accumulated in the burrow and entrance. During this process, the forelegs were used in unison and the wings were held flat on the dorsum. Females distributed the removed soil to distances of 25–40 mm, raking it in various directions with the forelegs. This activity resulted in leveling the sand in front of the entrance. After leveling the sand sufficiently females entered their burrows, turned around, and reappeared headfirst in the opening. Facing away from the burrow, they flung sand grains and other surface debris into the opening with the forelegs as they exited. Four such temporary closures of the nest entrance lasted 1.3–2.3 min in duration. Length of time spent to excavate a burrow and make a temporary closure of the nest entrance was 39 and 50 min, respectively, in two examples.

### Orientation flight

After making the initial temporary closure of the entrance, females made one or more orientation flights above the nest area. One wasp made three brief orientation flights in succession, landing on the ground surface near the nest between each flight. Another female made a single orientation flight that extended to a distance of 25 cm from the entrance before flying away. A third wasp flew in a figure eight configuration, beginning 5 cm from the filled entrance and ending 90 cm away before flying upward into the forest canopy to hunt for prey. Two extensive orientation flights lasted 4 and 7 min, respectively, including landings and pauses.

### Prey transport and nest entry

No ground transport of prey was observed, although some cockroaches weighed two to three times the weight of the wasp. All 69 examples of prey transport indicated the prey is carried in flight from the forest canopy to the nest. Five females averaged 22.7 min (range = 8–45, n = 6) between consecutive returns to the nest with partly paralyzed cockroaches. Three wasps averaged 6.8 min (range = 1.5–11.0, n = 4) between consecutive returns to the nest without prey.

**Figure 1.**
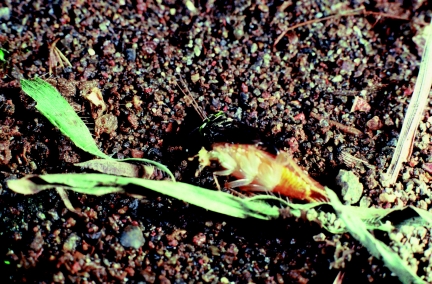
*Tachysphex inconspicuus* female removing temporary sand closure from nest entrance with forelegs while holding *Chorisoneura* prey to one side, perhaps with a hindleg, during nest entry. High quality figures are available online.

At the nest a female removed the temporary closure of the entrance using her forelegs and, retaining the grasp of the cockroach, entered the burrow rather rapidly without releasing the prey (Figure 1). The exact grasp of the cockroach during entry was not ascertained, although the field notes and one photograph indicate it was carried into the burrow to one side of the wasp and probably held by the hindleg on that side. After entering the burrow and placing the prey in the cell, a female reappeared in her entrance and re-closed the nest with soil and surface debris in about 2 min (n = 3). Wasps then flew almost straight upward into the forest canopy to search for additional prey.

### Final closure

Three final closures of completed nests were observed. The wasps raked sand backward with the forelegs into their burrows, coming out of the entrance repeatedly to obtain additional sand and debris from the ground surface. Each backward entry into the burrow with sand was followed by quick bursts of tamping the soil in place at the bottom with the pygidial plate (terminal dorsal sclerite of the abdomen). Most tamping of the soil fill at the bottom of the burrow occurred inside the nest and out of sight. One wasp dug a shallow depression in the sand surface next to her entrance in order to obtain additional soil for the fill. Two final closures took 13.5 and 15.5 min, respectively.

### Nest structure and dimensions

Females excavated short, shallow one- and two-celled nests, although it is not known whether all of the nests were finished. Twenty-two of 24(91.7%) nests were onecelled and two (8.3%) were two-celled. The burrows, 4–5 mm in diameter, entered the soil obliquely at 45–55° angles with the surface, leveled off, and went rather straight. One twocelled nest had cells at depths of 18 and 26 mm. Another two-celled nest had cells at depths of about 15 (2^nd^ cell) and 30 mm (1^st^ cell) beneath the surface. In the latter nest, the 2^nd^ cell was more inclined than the 1^st^ cell. Soil from the excavations leading to the second cells was probably used to fill the burrows leading to the first cells as females did not walk onto the surface to obtain additional soil. Burrow lengths, including cell lengths, of three nests were 28–45 mm. The elongate-oval cells were 7–8 mm high, 8–9 mm wide, and 15–18 mm long (n = 4).

### Prey

Females preyed exclusively on small cockroaches belonging to the subfamily Pseudophyllodromiinae (family Blattellidae). Identified prey consisted of 1 adult of *Riatia fulgida* (Saussure) and 59 adults and 9 (13.0%) nymphs belonging to 11 species of *Chorisoneura* (Table 1). Five of the 11 species (45.5%) were undescribed, representing 24 of 60 adults (Table 1). Thirtyeight of the 60 (63.3%) adults were female and 22 (36.7%) were male. Six additional prey from three nests were not identified to species.

**Table 1.**
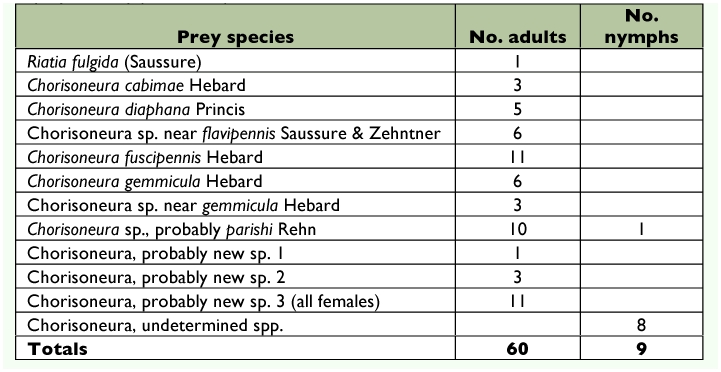
Species of prey of *Tachysphex inconspicuus*

**Figure 2.**
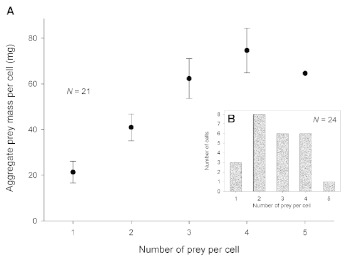
(A) Relationship between number of prey per cell and aggregate prey mass (wet weight) per cell (mean ± SEM) in 21 completed cells. Overall, the aggregate prey mass increased linearly with the number of prey per cell. (B) Frequency distribution of number of prey per cell in 24 completed cells. High quality figures are available online.

Five of the six were adult females and one adult male, bringing the total sex ratio to 65.2% (43) females and 34.8% (23) males.

**Figure 3.**
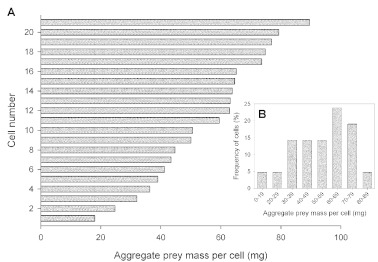
(A) Distribution of aggregate prey mass (wet weight) per cell across 21 completed cells. The cells are sorted in ascending order of aggregate prey mass. (B) Frequency distribution of aggregate prey mass per cell in 21 completed cells. High quality figures are available online.

The number of prey per fully provisioned cell ranged from 1 to 5 (mean = 2.75 ± 0.23 (SEM), n = 24 cells). Twenty of 24 cells (83.3%) held 2, 3, or 4 prey (Figure 2). The aggregate prey mass per cell increased linearly with the number of prey per cell (Figure 2). The distribution of aggregate prey mass per cell appeared normal (Figure 3), and it is assumed that cells containing 1 or 2 prey would have produced male wasps and those with 4 or 5 prey, female wasps.

Some adult cockroaches were slightly longer than *T. inconspicuus*, but weighed (wet) two to three times as much. Adult prey measured 5.5–10.0 mm in body length with an average of about 8 mm. Adult female cockroaches weighed, on average, 21.7 ± 0.76 mg (range = 10.6–29.8, n = 36), adult males averaged 17.8 ± 1.27 mg (range = 9.9–30.5, n = 20), and nymphs averaged 12.4 ± 0.86 mg (range = 8.7–16.8, n = 9). There were highly significant differences among the weights (wet) of the three life stages (ANOVA, *F* = 15.299, df = 62, 2, *P* < 0.0001). The average weight (wet) of *Tachysphex* females with a body length of 7 mm approximates 10 mg (Kurczewski FE, personal observation).

The aggregate prey weights of 21 cells for which mass data were available ranged from 18.0 to 89.6 mg with an average of 54.9 ± 4.18 mg. Cells that would have presumably produced female wasps (4–5 prey) weighed 63.1–89.6 mg (mean = 73.2 ± 3.65, n = 7). Cells that may have given rise to male wasps (1–2 prey) weighed 18.0–50.0 mg (mean = 36.6 ± 3.39, n = 9, Figure 3). Most of the cockroaches were positioned in the cell head inward and ventral side upward or on the side.

### Mutilation

**Figure 4.**
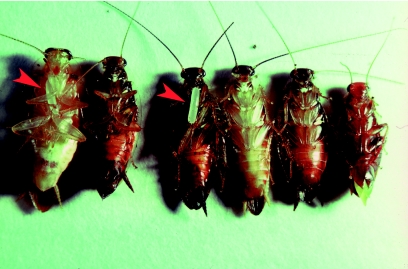
Contents of two-celled nest of *Tachysphex inconspicuus* with two and four prey, respectively, showing wasp eggs (arrows) affixed to forecoxal coria of 1^st^ and 3^rd^ cockroaches from left and extending longitudinally posteriad across thoracic venters. *Riatia fulgida* is on the far right; the other five cockroaches are *Chorisoneura* species. Note amputated antenna segments on some of the prey. High quality figures are available online.

Accidental mutilation or purposeful amputation of the prey's antenna and leg segments was evident on some of the recently captured cockroaches (Figure 4). Accidental mutilation may have occurred during prey capture. Purposeful amputation may have preceded the wasp feeding on the cockroaches' hemolymph at the site of the disjunction.

### Egg

The *T. inconspicuus* egg was usually laid on a cockroach at the bottom of the cell, near the back end. The prey to which the egg was affixed was invariably placed ventral side upward or tilted slightly on its side. The cockroach to which the egg was attached was the heaviest individual in 13 of 16 cells (81.3%), second heaviest (2 of 16, 12.5%), or lightest prey (1 of 16, 6.3%). Of the 19 cells for which both aggregate prey mass and a wasp egg were available, two fully provisioned cells held only a single prey. Including all cells for which aggregate prey mass was unavailable, 18 of 22 eggs (81.8%) were laid on female cockroaches, 3 of 22 eggs (13.6%) on male cockroaches, and 1 of 22 eggs (4.5%) on a nymphal cockroach. Oviposition on the lightest cockroach (nymph) occurred in an unusual cell provisioned with 5 prey: 3 adult males (11.0, 12.2, 13.0 mg), 1 adult female (18.3 mg), and 1 nymph (10.1 mg).

**Figure 5.**
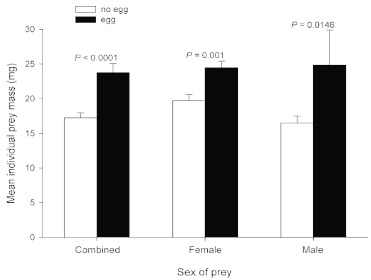
Relationship between mass of individual prey (wet weight ± SEM) bearing *Tachysphex inconspicuus* egg and other prey in cell, sorted by sex of prey. Prey on which wasp oviposited was significantly heavier than other prey in cell. High quality figures are available online.

Egg-bearing cockroaches weighed 10.1–30.5 mg (mean = 23.8 ± 1.26, n =19). Other prey in the cells weighed 8.7–26.7 mg (mean = 17.2 ± 0.71, n = 44). Egg-bearing prey averaged significantly heavier than the mean weight of other prey in the same cells (*t* = 5.0241, n = 17, *P* = 0.0001) or in comparison to each prey within the cell (*t* = 4.2330, n = 32, *P* = 0.0002). Similarly, eggs were laid on the largest female in comparison to non-eggbearing females and on the largest male in comparison to non-egg-bearing males (Figure 5).

The *T. inconspicuus* egg was affixed to the soft intersegmental membrane surrounding the base of a prey's forecoxa by the less tapered distal end (Figure 4). The proximal end extended longitudinally posteriad, across the thoracic venter of the cockroach. Two such eggs were slightly curved, sausage-shaped, white or ivory, and measured (live) 1.962– 2.191 mm long and 0.392–0.491 mm wide at the middle. One of the eggs weighed 0.3 mg.

### Enemies

The nesting activities of the *T. inconspicuus* were interrupted frequently by several species of foraging ants of various sizes. One provisioning female disturbed by ants flew onto a leaf, 10 cm away, and released her cockroach. She flew back to her nest, faced off an ant, examined the plugged entrance, and flew away. Within minutes, other ants discovered the paralyzed cockroach and carried it to their nest 7 cm away.

A small ctenid or sparassid spider pounced on one nesting wasp. But, it immediately jumped off. A second pounce by the spider in the direction of the wasp caused her to fly away. The wasp later landed behind the spider causing it to jump away!

## Discussion

*Tachysphex inconspicuus, T. iridipennis*, and *T. alayoi* nest through much of the year in the tropics in synchrony with alternating wet and dry seasons. *Tachysphex inconspicuus* and *T. iridipennis* have been collected in nearly every month, including December and January, according to specimens in insect museums (Brady S 2009, personal observation; Pulawski WJ 2009, personal observation). *Tachysphex alayoi* nests from early March through December in the Caribbean Region (Genaro JA 2009, personal observation). Many species of *Tachysphex* produce successive generations in warmer climates at approximately six weeks intervals (Kurczewski FE, personal observation), leaving open the possibility for multiple generations per year in the Caribbean and Neotropical Regions.

Nearly all prey records for species in the *obscuripennis* group are for the family Blattellidae, regardless of geographic region (Adlerz 1904; Bouwman 1916; Buys 2006, 2007; Callan 1954, 1993; Evans et al. 1976; Elliott et al. 1979; Genaro 2004; Grandi 1928, 1961; Krombein and Pulawski 1994; Maneval 1932; Rau 1933; Vesey-FitzGerald 1956; Williams 1932, 1945; G. Beccaloni 2009, personal observation). Records from the Palearctic Region include the subfamily Ectobiinae of the family Blattellidae. Among the American species, *T. inconspicuus* apparently stocks its cells with different prey than *T. alayoi* or *T. iridipennis. Tachysphex inconspicuus* preys on “canopy indicators” (Fisk 1983) belonging to the genera *Chorisoneura* and *Riatia*, both members of the subfamily Pseudophyllodromiinae (Blattellidae) (Buys 2006, 2007; Callan 1954, 1993; Pulawski 1974, 1988; this study). Females fly into the canopy of the tropical wet forest to search for such species and fly from there to their nests with partly paralyzed prey individuals. This prey specialization is underlined by the relatively large percentage (45.5%) of undescribed species provisioned by *T. inconspicuus.*

*Tachysphex iridipennis* captures blattellids in the genera *Cariblatta, Ischnoptera*, and *Euthlastoblatta* all in the Pseudophyllodromiinae, and perhaps *Anaplecta* in the Anaplectinae (Pulawski 1988; Rau 1933; Vesey-FitzGerald 1956). *Tachysphex alayoi* preys upon blattellids in the genera *Cariblatta* and *Symploce* (Blattellinae) (Elliott et al. 1979, Genaro 2004). Females of *T. alayoi* were observed flying from higher vegetation to the ground with partly paralyzed cockroaches (Elliott et al. 1979).

Nearly all observations of prey transport in the *obscuripennis* group, including all 69 examples observed in *T. inconspicuus*, indicate that the mainly small cockroaches are carried in flight (Buys 2007; Elliott et al. 1979; Evans et. al. 1976; Maneval 1932; Rau 1933; Williams 1945). Krombein and Pulawski (1994) reported a female of *T. drymobius* Pulawski in Sri Lanka, 9.5 mm long, flying with an adult male cockroach, 14.0 mm long. This size disparity should have necessitated ground transport. Elliott et al. (1979) noted females of *T. alayoi* flying with prey 1.98–2.76 times their weight.

The structural adaptations that distinguish species of the *obscuripennis* group from other *Tachysphex* may allow them to fly with larger prey. In addition to the tarsal modifications, *T. inconspicuus* females have a robust thorax for an extensive internal flight musculature and long, broad wings with a greater total surface area than many similar-sized species in the *pompiliformis* group that practice ground transport. In this regard, species in the *obscuripennis* group resemble species in the *terminatus* group that carry their prey in flight (Kurczewski 2009).

The description of females of *T. inconspicuus* retaining their grasp of the prey with the mandibles and a hindleg, removing the fill from a temporarily closed entrance with the forelegs, and quickly entering the burrow represents atypical behavior among *Tachysphex* species (Buys 2007; Kurczewski 2010). *Tachysphex albocinctus* in the *albocinctus* group, a mantid-hunter (Asis et al. 1989), and *T. mediterraneus* in the *plicosus* group, a tree-cricket-hunter (Ferton 1923), exhibit similar nest entry behavior. All three species nest in sandy soils, level the sand removed from burrow excavation, and make a temporary closure of the nest entrance prior to hunting for prey. Most *Tachysphex* release the prey on the ground before entering their nest.

The field observations of this study and Figure 1 indicate that *T. inconspicuus* prey are carried to one side of the wasp and perhaps grasped by a hindleg. Holding the cockroach to one side may enable the female to remove the soil from the entrance with the forelegs unimpeded, thereby ensuring a more rapid entry. Such a manner of entry would have positive implications when considering the endless encounters and interactions at the nest between the wasps and foraging ants and cleptoparasitic flies, respectively (Buys 2007, this study).

*Tachysphex inconspicuus* has a unique combination of structural adaptations on the hindlegs that may be connected with holding the cockroach to one side during nest entry: 1) basal tooth on hindcoxa, 2) slender hindfemur, 3) asymmetrical hindtarsal claws, 4) hindtarsomere IV undulate apicoventrally (its membrane exposed) 5) pronounced mat-like vestiture on ventral hindtarsomere V (Pulawski 1988). Neither *T. iridipennis* nor *T. alayoi* has this combination of hindleg adaptations.

The nests of species in the *obscuripennis* group, including *T. inconspicuus*, are rather short and shallow (Buys 2007; Evans et al. 1976; Genaro 2004; Maneval 1932; Williams 1945; this study). Nests in loose sandy soil tend to be longer and deeper than nests in compact sandy soil (Buys 2007). Most nests completed by species in the *obscuripennis* group are single-celled (Buys 2007; Evans et al. 1976; Genaro 2004; this study), but, rarely, two cells are excavated per nest in *T. fanuiensis* from New Caledonia and Australia (Williams 1945) and *T. inconspicuus* (this study).

The number of prey cockroaches per fully provisioned cell in species in the *obscuripennis* group ranges from 1 (*T. inconspicuus*, this study) to 6 or 7 (*T. fanuiensis*, Williams 1945). More species complete cells with 2 prey than any other number: *T. depressiventris* (Evans et al. 1976), *T. inconspicuus* (Buys 2007; this study), *T. alayoi* (Genaro 2004), *T. fanuiensis* (Williams 1932), and *T. obscuripennis* (Maneval 1932). Completed cells may contain a mix of adult and nymphal cockroaches. There is a preponderance of adult females among the cell provisions of *T. inconspicuus*, but it is the only species in the group that has been studied sufficiently to draw any conclusions. Nevertheless, sexually reproducing cockroach species generally produce a 1:1 sex ratio (Bell et al. 2007), so the preponderance of adult females and significant under-representation of nymphs suggest that the wasps specifically hunt for the largest *Riatia* and *Chorisoneura.* Moreover, four of the prey females carried oothecae. Because females in this physiological state generally forage and feed less than vitellogenic females, *T. inconspicuus* females may be adept at finding such females in their hiding places.

Hypothetically, cells containing one or two prey and weighing half as much or less than cells with four or five prey are likely to produce male wasps. Cells holding four or five prey and weighing about twice as much, or more than cells with one or two prey probably produce female wasps. Aggregate prey weights of fully provisioned cells indicate that prospective female cells weigh, on average, about twice as much as prospective male cells. Brockmann and Grafen (1989) demonstrated that females of *Trypoxylon politum* (Crabronidae) stock male cells with less biomass of spiders than female cells. Coville et al. (2000) found that male cells of *Trypoxylon vagulum* had less prey biomass than female cells, although the numbers of prey spiders were not significantly different.

The eggs of species in the *obscuripennis* group from all geographic regions are laid obliquely or longitudinally posteriad instead of transversely across the prey's thoracic venter, as in the majority of *Tachysphex* species (Evans et al. 1976; Genaro 2004; Grandi 1961; Maneval 1932; Williams 1945; this study). Such a placement represents genus-atypical behavior and a possible diagnostic taxonomic character for this species group (Evans et al. 1976; Kurczewski 2010).

The majority of females of *T. inconspicuus* laid their eggs on the heaviest cockroaches in the cells, in most cases adult females. The wasps show an uncanny ability to make oviposition choices based on the size, weight, or mass of the prey individuals. The example in which a female laid her egg on the smallest prey in the cell (10.1 mg) occurred in the only cell with five cockroaches, the maximal number of prey per cell. Perhaps this wasp had so little space within the cell in which to maneuver she laid her egg indiscriminately on the closest individual.

Mutilation of leg or antennal segments of the cockroach occurs in *T. depressiventris* (Evans et al. 1976), *T. obscuripennis* (Grandi 1961), *T. alayoi* (Genaro 2004), and *T. inconspicuus* (this study) and may characterize all species in the *obscuripennis* group. In T. *depressiventris*, one or more legs are sometimes missing, perhaps related to malaxation of the prey by the wasp for purposes of feeding on hemolymph or possibly the struggle that ensued during capture of the cockroach (Evans et al. 1976).
